# Artificial intelligence in blood donor management: A narrative review

**DOI:** 10.1111/vox.70141

**Published:** 2025-10-28

**Authors:** Maha A. Badawi

**Affiliations:** ^1^ Department of Hematology, Faculty of Medicine King Abdulaziz University Jeddah Saudi Arabia; ^2^ Blood Transfusion Services Unit King Abdulaziz University Hospital Jeddah Saudi Arabia

**Keywords:** artificial intelligence, blood donation, blood donor management, chatbot, machine learning, robotic process automation

## Abstract

Blood transfusions are vital in health care, yet maintaining an adequate and safe blood supply remains a significant challenge. To address blood donation–associated challenges, this review explores how integrating artificial intelligence (AI) technologies can improve donor recruitment, retention and management. For instance, robotic process automation can streamline repetitive administrative tasks, allowing staff to focus on more critical activities and improving efficiency. When augmented with AI techniques such as machine learning (ML) and natural language processing, it transitions from static rule‐based automation to intelligent process automation. This combination enables dynamic decision making, handling unstructured data and optimizing workflows, thus extending its role in improving efficiency and decision making in donor management. ML algorithms can analyse large datasets to predict future donation patterns, identify donor behaviour trends and forecast blood demand more accurately. By applying these predictive models, blood banks can plan more effectively, avoid shortages and precisely target recruitment efforts. Additionally, AI‐driven chatbots are gaining traction as a tool for improving communication with potential and existing donors, ultimately fostering better retention rates. Beyond routine donor management, AI also shows promise in supporting rare donor identification and targeted engagement strategies. While these innovations hold great potential, their implementation faces challenges such as data availability and quality, ethical issues concerning AI utilization, the necessity for clinical and technical expertise, a robust infrastructure, environmental impact and cybersecurity risks. Addressing these issues through practical strategies and thoughtful integration will be the key to ensuring the responsible, effective and sustainable adoption of AI in blood banking systems.

**Highlights**

- Artificial intelligence (AI) integration into blood donor management systems can potentially improve operational efficiency and enhance donor engagement and retention.
- AI tools such as robotic process automation can streamline routine processes and repetitive tasks, machine learning algorithms can analyse donor behaviour and predict patterns and AI‐driven chatbots can improve communication and engagement with donors.
- Key challenges with AI implementation in blood donor management include data limitations, ethical concerns, the need for clinical and technical expertise, environmental impact and cybersecurity risks. Addressing these challenges is essential for the successful and responsible application of AI in health care.

## INTRODUCTION

Blood transfusions are crucial in both routine and emergency healthcare settings, with donations being the sole source of blood supply. Nevertheless, significant challenges remain in maintaining a sufficient and safe blood supply, particularly in developing and transitional countries. The demand for blood transfusions outgrows the supply [1], and blood donation rates remain low overall [1, 2].

A significant challenge is that family and replacement donors remain the primary source of blood donations in the Eastern Mediterranean region, accounting for 49.4% of all blood donations. This heavy reliance on family and replacement donors leads to inconsistent and insufficient blood supplies [2]. Because these donations are often driven by urgent personal needs rather than a steady commitment, hospitals face challenges in maintaining a reliable blood supply, especially in emergencies or for regular transfusions [1].

To address the challenges associated with relying on family and replacement donors and the blood shortages experienced by blood banks worldwide, a transformative approach that integrates marketing principles with artificial intelligence (AI) technology is beneficial. This approach can improve donor recruitment, retention and management. By implementing a marketing‐focused strategy, we can effectively enhance the appeal of the blood donation experience, making it more attractive to potential donors [3, 4, 5, 6, 7, 8]. Lessons from successful marketing campaigns in blood donation or other sectors can be adapted to raise awareness, improve the perceived value of donating and build donor loyalty [4, 6, 9]. Integrating AI into these efforts can refine marketing campaigns by analysing donor behaviour, predicting donation patterns and personalizing outreach efforts. This approach can create a more dynamic and effective donor management system, ensuring a steady and reliable blood supply. AI‐driven insights can help blood banks to understand the motivations and barriers influencing donor behaviour, allowing them to design more compelling marketing campaigns that resonate with different donor profiles.

This review paper will provide an overview of recent literature on the use of AI in blood banks, focusing on its application in blood donor management. We will examine various AI techniques that contribute to process improvement and enhanced donor experiences, including the automation of tasks through robotic process automation (RPA) and big data analysis using machine learning (ML) for predictions. The paper will also cover AI‐driven chatbots for improved donor communication, as well as AI applications in rare donor identification and management. In addition, the review will address the challenges associated with implementing AI in these settings and conclude with practical considerations for the successful and responsible adoption of these technologies.

## AI IN ENHANCING BLOOD DONOR MANAGEMENT: THE LITERATURE

**FIGURE 1:**
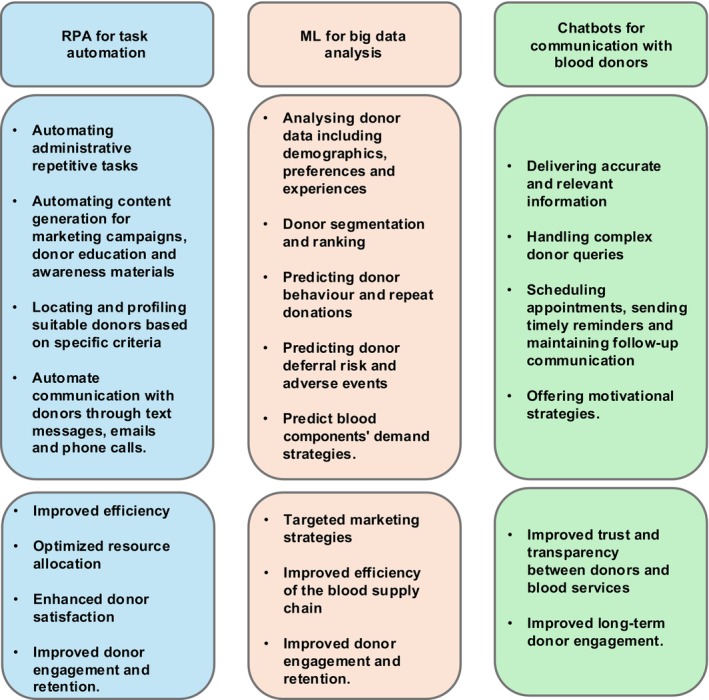
Application of artificial intelligence (AI) in blood donor management: Integration and gained benefits. ML, machine learning; RPA, robotic process automation.

The application of AI in blood donor management has the potential to significantly improve the efficiency and effectiveness of blood donation processes [10]. AI technologies such as RPA can automate repetitive tasks [11, 12, 13], while ML techniques can analyse vast datasets to predict demand and optimize donor recruitment [12, 13]. AI‐powered chatbots streamline communication with donors [12, 13, 14]. These advancements help blood banks to improve operational efficiency and foster stronger connections with donors through personalized, data‐driven interactions (Figure 1).

### RPA for efficient blood donor management

RPA refers to software tools that mimic human actions to perform manual, routine, rule‐based tasks across various systems [11]. It works by replicating a human's actions on a computer and interacting with digital systems to complete tasks such as processing forms, updating databases or extracting information from various sources [12, 13]. In blood donor management, RPA can automate repetitive activities such as entering donor information, analysing blood bank data and cleaning datasets for quality assurance. It can also assist in processing blood donor registrations, ensuring accurate records storage in the system and eliminating data inconsistencies through automated validation processes [11]. Moreover, RPA can handle simple queries, allowing staff to focus on more serious responsibilities such as strategy and donor engagement [12, 15]. Essentially, RPA optimizes and automates operational workflows, replacing manual repetitive tasks, eliminating unnecessary steps and improving overall efficiency [16]. While traditional RPA employs rule‐based automation to execute repetitive tasks, its integration with AI techniques, such as predictive analytics and natural language processing (NLP), transforms it into intelligent process automation (IPA). This hybrid approach combines knowledge‐driven methods with data‐driven AI, enabling systems to adapt dynamically, learn from historical data and handle more complex scenarios [17]. For example, AI‐enhanced RPA in blood donor management can analyse donor profiles, predict appointment attendance and recommend targeted outreach strategies.

Saeed et al. [18] have proposed an automated bioinformatics AI software system that replaces manual data entry in blood banks, handling donor registration, recording donations, managing blood product inventory and issuing blood. Another example is the automated appointment scheduling system, which can reduce no‐shows and optimize resource allocation by providing real‐time availability and reminders to donors, potentially reducing missed appointments [19]. Additionally, RPA bots can automate the entry and duplication of information in electronic health records, reducing clerical work for staff and minimizing human error [20]. RPA software can also streamline blood donor eligibility pre‐screening to quickly analyse donor health data, reducing the need for lengthy questionnaires [19]. AI can access past donation records and health data to avoid repetitive questions for regular donors with consistent histories. Additionally, AI systems can cross‐check travel history with epidemiological data, assess risks for infectious diseases and prompt additional questions only if a risk is detected, while skipping unnecessary questions. This ensures blood safety, improves efficiency and minimizes superfluous deferrals [19]. In their study, Bin et al. [21] demonstrated the potential of AI solutions in automating administrative tasks within an urgent care setting, such as medical care registration. By automating this process, the digital solution significantly reduced patient waiting times by nearly 12 min and saved approximately 2500 h over 12 months [21]. Another study from Taiwan described an RPA method to streamline the medical expense claims processing at a local hospital, decreasing the total process time by 380 min and increasing process cycle efficiency from 69.07% to 95.54%. These improvements are expected to enhance customer satisfaction and increase internal satisfaction while freeing administrative staff to focus on more critical tasks [16, 21]. These examples highlight the efficiency gains and time savings that AI‐driven automation could similarly bring to blood donor system management.

In addition to handling administrative tasks, RPA can automate content generation for marketing campaigns, donor education and awareness materials [22]. AI‐powered content creation tools can generate written materials, videos and social media posts to engage donors and encourage repeat donations. These tools can customize content to suit different donor demographics and profiles, ensuring that the information is relevant, timely and effective [13, 19].

Furthermore, RPA can streamline locating and profiling suitable donors based on specific criteria, such as blood type, availability and proximity to a donation centre. As such, RPA systems can rapidly mobilize donors in emergencies and focus outreach efforts on those most likely to respond positively [10, 12]. This system allows for faster decision making and better resource management, particularly during critical periods of blood shortages.

Finally, RPA can automate donor communication through text messages, emails, and phone calls. It sends reminders for upcoming appointments and post‐donation follow‐ups and provides information about future donation opportunities [19, 22]. Chinnaswamy et al. [23] describe an intelligent system for automating blood donation processes, which employs short message service (SMS) technology to send automated notifications when blood stock levels fall below specific thresholds. Depending on the urgency of the bloodstock deficit, different groups of eligible donors are notified. The notification system is designed to prevent blood shortages and minimize wastage by adjusting the number of donors contacted based on the severity of the stock deficit, ensuring timely replenishment of supplies [23].

By automating routine tasks, RPA allows staff to dedicate more time to complex, high‐level activities such as donor relationship management, strategic planning and improving service quality [12, 22]. Staff can shift their attention from repetitive administrative tasks to functions that require human decision making, empathy and creativity [11, 20]. Additionally, RPA tools significantly reduce the potential for human error by ensuring that repetitive tasks are completed accurately and consistently [11, 20]. This is critical in blood banking, where mistakes in donor records, inventory management or appointment scheduling can have serious consequences. RPA also improves accuracy and standardizes processes [11], leading to more consistent outcomes and higher quality results.

### ML for optimized blood donor management

As a subset of AI, ML allows for advanced and sophisticated analysis of large datasets, enabling the prediction of trends, optimization of strategies and streamlining of system management [24, 25]. These technologies can potentially transform blood donor management by ensuring a stable and efficient blood supply [10, 26]. They introduce new methodologies by predicting blood demand and donor behaviour, personalizing communication and optimizing recruitment and retention strategies [13, 27, 28]. Additionally, they allow for more effective inventory management, reducing waste and mitigating potential shortages [26, 28, 29].

#### ML application in donor behaviour prediction

One of the key applications of ML in blood donation is the prediction of donor behaviour based on historical donation data. ML can identify patterns and trends in donor responses and predict a donor's likelihood of future contributions, enabling refined and targeted recruitment strategies [30, 31]. Studies by Martin‐Santana [32] and Romero‐Domínguez [33] used ML techniques to categorize donors and non‐donors based on various motivations and barriers. They proposed targeted marketing strategies to address specific donor concerns, ultimately improving recruitment and retention efforts [32, 33]. Similarly, Pabreja et al. [34] employed ML algorithms to classify university students as donors or non‐donors, identifying critical factors such as the time taken for the process, having a companion and feelings of reward as significant determinants of donation decisions. These insights help improve recruitment strategies and organize more efficient blood donation campaigns among youth [34].

Wu et al. [35] used ML algorithms to analyse large‐scale historical donation data to predict and rank donors' willingness to donate during the coronavirus pandemic. They processed SMS recruitment data and donation records from previous years to generate personalized scores for each donor, indicating the likelihood of future donations. This ranking system allowed blood agencies to develop targeted recruitment strategies, improving the overall effectiveness of donor outreach and ensuring a stable blood supply during the pandemic [35]. Similarly, Gammon et al. [36] analysed historical anonymized donation data from Italy, Singapore and the United States to determine the likelihood of donor return based on predictive models. Despite donation declines during COVID‐19, the models consistently identified donors most likely to return, validating their effectiveness [36]. Research by Salazar‐Concha and Ramírez‐Correa [37] aimed to predict the intention to repeat blood donations using a decision tree technique based on a limited number of attributes related to blood donation intention, while Alajrami et al. [38] proposed an artificial neural network model to predict whether a person will donate blood based on previous donation behaviour. Both models demonstrated very high prediction accuracy. Cloutier et al. [27] used random forest models to analyse sociodemographic, marketing and adverse event data to predict the donation frequency of young whole‐blood donors. They concluded that using ML and large datasets provided a more accurate prediction of donor behaviour than conventional methods, enabling better donor retention strategies. Ben Elmir et al. [26] describe an 11% increase in collected blood volumes and a 20% reduction in wastage by targeting regular donors based on their past behaviour using AI‐driven decision support tools. Finally, AI can predict seasonal donation patterns and regional health issues, allowing blood banks to proactively adjust their campaigns to prevent shortages [19].

#### ML application in deferral risk prediction

AI‐driven prediction models can improve the donor recruitment process by identifying donors at risk of deferral and optimizing donation timing, thus ensuring that only those likely to be eligible are invited. Such models could prevent the majority of on‐site deferrals, ultimately enhancing donor motivation and optimizing the blood supply [39]. Furthermore, ML models can identify donors at risk of iron deficiency by analysing trends in haemoglobin and ferritin levels, allowing for pre‐emptive adjustments to donation schedules. Studies have shown that ML‐based approaches could prevent the majority of on‐site deferrals due to low haemoglobin, ensuring that only eligible donors are invited [39].

#### ML application for personalized outreach strategies

AI‐driven systems enhance personalized communication strategies, which is essential for improving donor engagement and retention. By leveraging predictive analytics, blood banks can determine the optimal timing for outreach, ensuring that the right message is delivered to the proper donor when they are most likely to contribute [19, 30, 40]. This data‐driven approach improves the relevance of interactions, leading to more effective and cost‐efficient donor retention efforts [10, 41]. Furthermore, ML improves social media–based recruitment by identifying relevant posts, such as those addressing the need for blood donations. AI can then match potential donors with nearby opportunities through platforms such as the Facebook Blood Donation feature, which links millions of donors with donation sites across multiple countries. This tool allows users to receive targeted notifications about nearby blood drives, ensuring they stay informed. [42]. These systems also enable personalized outreach, ensuring that individuals who have shown interest in donating are promptly informed of urgent needs, thereby significantly increasing the likelihood of their participation [43].

Furthermore, donor segmentation allows blood banks to create customized outreach strategies that resonate with different demographic groups, such as personalized invitations [39] or targeted social media campaigns at specific age groups [19]. This segmentation helps in building stronger relationships with donors and increases donation rates [12]. Integrating ML into customer relationship management systems can streamline communications with donors, creating a feedback loop where engagement strategies are continuously refined based on real‐time data [13]. This fosters a sense of belonging among donors, increasing the likelihood of repeat donations. Offering exclusive invitations to highly engaged donors strengthens donor–blood bank connections, leading to deeper, long‐term loyalty [19].

ML can help address factors contributing to donor attrition by identifying reasons such as negative experiences or lack of recognition. Tailored outreach campaigns based on these insights can significantly reduce donor dropout rates and increase donation frequency [30]. By integrating AI‐driven insights, blood banks can improve operational efficiency and create meaningful, personalized interactions that encourage sustained donor engagement.

#### ML application for ensuring donor safety

Lastly, ML can also play a pivotal role in enhancing donor safety. ML models can predict adverse reactions, such as fainting or vasovagal episodes, during donations by analysing donor‐specific factors and environmental conditions such as local weather [44, 45]. These models have demonstrated high predictive accuracy and can allow blood centres to take preventative steps, such as pre‐donation hydration or targeted monitoring of at‐risk donors [44]. This proactive approach minimizes discomfort and helps reduce negative experiences associated with blood donation. In addition, AI can help analyse measured donor haemoglobin and ferritin to optimize donation intervals for specific donor groups, thereby preserving their iron levels [39]. By leveraging similar AI‐driven predictive models, blood banks could analyse trends in individual donors' iron levels, identify patterns over time, predict the risk of iron deficiency and proactively adjust donation schedules to ensure donor safety. This personalized approach ensures that donors with sufficient iron stores can donate more frequently while those at risk receive extended recovery periods. By integrating additional factors such as demographics, body mass index (BMI), diet and genetic markers, AI can further refine predictions and tailor donation intervals to individual needs. This not only reduces deferral rates by ensuring that donors are invited at optimal times but also enhances donor retention by minimizing unexpected deferrals and supporting long‐term donor health.

### Chatbots for communication with blood donors

AI‐powered conversational agents, such as virtual agents, chatbots or embodied conversational agents, have significantly improved communication in various industries, including health care and blood donation. AI‐powered chatbots use speech recognition and NLP to interact with users through text or voice, allowing for dynamic, personalized and flexible interactions [46]. AI‐driven chatbots have emerged as powerful tools in blood donor management, enhancing efficiency and improving donor experiences. They provide real‐time feedback and sentiment analysis, helping blood banks to promptly address donor concerns, ensuring smoother donation processes and fostering trust [19]. They can also offer accurate and accessible information about blood donation, dispel misconceptions and provide guidance at any stage of the donation process [47].

Furthermore, AI's flexibility in processing natural language queries enables chatbots to handle complex donor inquiries, even when faced with incomplete or incorrect information [48]. These systems reduce the workload for human operators by automating repetitive tasks and contribute to donor retention by providing immediate support for issues that may arise post donation [19]. The potential for AI chatbots to manage over 85% of interactions without human intervention underscores their transformative impact on donor engagement, specifically in blood donor management [22].

Müller and Reuter‐Oppermann have conducted significant research on chatbots in blood donation services [49]. They have proposed an app‐integrated chatbot that offers donors comprehensive features and a carefully selected set of social cues, enhancing blood donor engagement. The researchers emphasize that AI‐driven chatbots should address potential barriers to increase blood donors' willingness to participate while providing perceived motivators. Müller and Reuter‐Oppermann design emphasizes three fundamental design principles. First, chatbots should provide organizational support by assisting donors by scheduling appointments, sending timely reminders and maintaining follow‐up communication. This helps reduce no‐shows and encourages continuous donor engagement. Second, chatbots should play a vital role in education, delivering accurate and user‐friendly information about the blood donation process and health benefits. They should address knowledge gaps and dispel misconceptions, which are key barriers for first‐time donors. By doing so, chatbots help build trust and transparency between donors and blood services. Third, chatbots should offer motivational strategies such as gamification, feedback loops and social recognition to increase long‐term donor engagement. They can offer rewards, milestones and emotional connections by highlighting the positive impact of donations, such as the number of lives saved [49]. This approach addresses critical issues in blood donor management by aligning chatbot functionality with donor behaviour insights, ensuring both short‐term and long‐term engagement [50]. Further, Müller and Reuter‐Oppermann [50] surveyed 371 potential blood donors from South Africa and Ghana to validate the effectiveness of these design principles. Participants offered positive feedback about the chatbot's ability to influence their donation behaviour.

### AI and rare donor management

Rare donor programmes face considerable challenges in identifying, retaining and mobilizing donors with uncommon blood phenotypes. These activities involve maintaining accurate registries, conducting molecular testing, managing frozen inventories and ensuring timely communication with donors during urgent needs [51]. AI offers valuable opportunities to enhance these processes. For instance, RPA can streamline repetitive tasks such as updating registries, cross‐checking molecular testing outputs or generating alerts when rare units require replenishment. At the same time, ML can accelerate the identification of rare phenotypes from high‐throughput testing and improve forecasting of demand by analysing utilization patterns alongside demographic data, thereby supporting more efficient inventory planning. Moreover, chatbots can play a role in sustaining engagement with rare donors through personalized communication, reminders of eligibility and rapid responses in times of need, reducing the burden on programme staff. Practical initiatives such as the Canadian Rare Blood Program [51] highlight the complexity of rare donor management and demonstrate how simulation modelling and molecular testing already contribute to these efforts; integrating RPA, ML and chatbots into such frameworks could further strengthen rare donor programmes globally by improving efficiency, engagement and preparedness.

## CHALLENGES AND CONSIDERATIONS IN THE APPLICATION OF AI IN BLOOD DONOR MANAGEMENT

Adopting AI in blood donor management offers numerous opportunities to improve efficiency and effectiveness. However, it also brings several challenges that require careful consideration (Table 1).

**TABLE 1:** Overview of challenges in the application of AI in blood donor management.

Challenges	Considerations
Data limitations	
Availability and quality of data	Complete and high‐quality data Continuous monitoring of AI models
Data exclusions and biases	
Limited generalizability of AI models	
Ethical use	
Data bias	Adherence to ethical principles Reliance on regulatory frameworks Fairness in donor representation Robust security measures Workforce planning
Data breach risks	
Transparency and accountability	
Job displacement	
Clinical and technical expertise	
Lack of familiarity with AI	Specialized education and training programmes Regular updates of AI models Systematic redesign of workflows
New data and evolving policies	
Computing power and infrastructure	
Significant computational resources	Piloting of AI projects Phased adoption Collaboration with third parties
Financial constraints	
Usability and user experience	
Resistance from potential users	Projects designed with user needs in mind Ongoing training and promotion of AI literacy
Environmental impact	
Increased energy demand	Investing in cutting‐edge hardware Improving data centres' efficiency Strategic selection of AI applications Accurate measurements of power consumption
Cybersecurity threats	
Increased risk of cyber attacks	In‐house development of AI models Adversarial training Continuous monitoring of AI behaviour

Abbreviation: AI, artificial intelligence.

### Data limitations

The success of ML models heavily relies on the availability and quality of data. Without sufficient and usable data, AI cannot accurately learn patterns or make predictions, which limits ML models' effectiveness [13]. Another critical issue is data exclusions. For instance, deferred donors or those with incomplete data records are often excluded from analyses, which introduces selection bias and reduces the generalizability of the models [35, 44]. Additionally, most ML models used in blood management have been trained on data from a single centre or a limited number of centres, which limits their applicability across broader and more diverse populations. As a result, the models' effectiveness diminishes when deployed in different settings [52].

### Ethical use of AI

The ethical implications of AI are a major concern in blood donor management, especially when it comes to bias, privacy and transparency. ML models are only as unbiased as the data on which they are trained; if these datasets reflect societal biases, the AI system may unintentionally perpetuate discriminatory practices based on race, gender or socioeconomic status [10]. Transparency in decision making is crucial, as donors and recipients must understand how decisions that affect them are made [10], and stakeholders should be accountable for such decisions [53]. Ensuring that AI systems adhere to ethical principles such as fairness, accountability, privacy and safety is vital for maintaining trust [53]. This is why regulatory frameworks, national or organizational [53, 54], are necessary to support and foster these principles.

Robust security measures are essential to protect sensitive personal information, as data breaches threaten donor privacy. Informed consent regarding how data will be used is equally important to safeguard the ethical implementation of AI [10].

Another significant concern is job displacement [11, 12]. As AI systems become more advanced, they can automate tasks traditionally performed by humans, raising fears of job losses [11]. Balancing AI's benefits with its potential to displace jobs will require thoughtful workforce planning, upskilling initiatives and creating new roles where human intelligence complements AI systems.

### Clinical and technical expertise

The successful integration of AI in health care relies on engaging various disciplines and providing continuous education and training for users to ensure the correct utilization of AI systems [55]. However, the lack of familiarity with AI among healthcare professionals remains a challenge, highlighting the importance of specialized education and training programmes to bridge this gap [56]. Organizations should tap into the expertise of skilled employees, such as data scientists, and promote a learning culture to implement new technologies effectively. Recruiting internal experts is also vital for ensuring successful adoption [11].

On the other hand, AI models must be regularly updated to incorporate new data and reflect evolving policies. Failure to do so can lead to inaccuracies and outdated predictions [39]. A systematic redesign of workflows may be necessary, reconsidering how tasks are allocated between human operators and AI systems to maximize their complementary strengths [11].

### Computing power and infrastructure

AI applications in blood donor management require significant computational resources, especially when processing large volumes of data. Organizations need centralized expertise to support AI application and maintenance and adequate infrastructure to ensure the smooth functioning of complex algorithms [11]. Implementing AI pilot projects allows for an incremental rollout, which helps in identifying potential issues before full‐scale deployment [11]. However, financial constraints often hinder AI adoption, as nonprofit and healthcare organizations may struggle to justify or secure funding for these advanced technologies [31].

### Usability and user experience

AI must be user‐friendly and intuitive to be successfully adopted in blood donor management. Systems that are difficult to use or understand may face resistance from potential users, limiting their efficacy [55]. Therefore, AI systems should be designed with user needs in mind to improve workflows rather than hinder them [11]. Ongoing training and promoting AI literacy within healthcare settings are essential for encouraging user acceptance and ensuring that AI tools are fully leveraged [25].

### Environmental impact

AI offers significant opportunities to enhance energy efficiency across multiple sectors and facilitate the energy transition; however, its energy use presents a paradox [57, 58]. The substantial energy demands of AI systems significantly add to global greenhouse gas emissions, especially given the high workloads of data centres [57, 58, 59]. Moreover, the increasing complexity of AI models leads to a dramatic rise in electricity consumption, resulting in further pressure on electrical grids and worsening global energy challenges [58]. To mitigate this impact, a multi‐faceted approach is necessary. This includes investing in cutting‐edge hardware, improving data centres' efficiency and strategically selecting AI applications to balance performance with environmental sustainability [57, 58, 59]. Accurate power consumption measurements are also vital for improving algorithms and creating energy‐efficient systems [57]. Fostering a sustainable future for AI requires not only technological advancements but also a strong commitment to ethical principles in the research and application of AI [58].

### Cybersecurity threats

Integrating AI into various systems increases the risk of cybersecurity threats by introducing new vulnerabilities and expanding the attack surface for malicious actors. AI systems rely heavily on large datasets and complex predictive models, which can be manipulated to alter system behaviour. For example, attackers can exploit AI's learning processes through techniques like data poisoning, where false data are injected into training sets, leading to inaccurate or harmful outputs. Similarly, predictive models can be tampered with, causing AI to make incorrect classifications or predictions, which could have serious consequences in sensitive sectors such as health care. These vulnerabilities, coupled with AI's reliance on vast and often sensitive datasets, make AI systems an attractive target for cyberattacks, necessitating stronger safeguards and more resilient cybersecurity strategies [60]. To enhance the reliability of AI in cybersecurity, Taddeo et al. [60] propose three strategies: in‐house development of AI models by reducing the risks associated with external cloud services that may expose models and data to attacks; incorporating adversarial training to improve system robustness and detect vulnerabilities; and implementing continuous monitoring through a control system to detect and address deviations in AI behaviour early, thus ensuring real‐time reliability.

Recently, ransomware attacks targeting blood supply systems in the United Kingdom and the United States have created major disruptions, resulting in blood shortages, cancelled appointments and delayed medical procedures. These incidents reveal the susceptibility of blood supply chains to cyber threats and emphasize the necessity for contingency planning to ensure a reliable supply during emergencies [61].

### Practical considerations and successful implementation

Integrating AI into blood donor management involves more than just technical aspects; it requires careful financial and operational planning. Although initial expenses for computing power, data systems and skilled staff may be high, cost–benefit analyses show significant long‐term benefits. These include streamlining administrative tasks through automation, increasing donor retention with personalized engagement and optimizing resource use with predictive analytics. Using phased approaches and pilot projects that demonstrate measurable results before full rollout can improve success rates. Collaborating with government agencies, technology providers and international groups can help offset costs and ensure sustainability and ethical standards.

Real‐world examples confirm that such investments deliver significant operational, financial and clinical benefits. Even simple tools, such as the SMS‐based alert system described by Chinnaswamy et al. [23], helped prevent shortages and reduce waste. Building on this, the Blood and Tissue Bank in Catalonia used an ML‐based system to optimize donor outreach, predict donor response and monitor performance through dashboards, resulting in more stable collections and increased contributions from key donor groups [62]. Similarly, Ben Elmir et al. [26] demonstrated scalability by achieving an 11% increase in collected blood volumes and a 20% reduction in wastage through predictive targeting of regular donors. In India, predictive algorithms have improved donor retention, reduced waste, enhanced blood‐type matching and minimized transfusion‐related adverse events—highlighting both efficiency and patient safety [63]. In Hamilton, Ontario, a hybrid ML and optimization model reduced red blood cell inventory by 38%, overall costs by 43% and ordering frequency by 63%—without increasing shortages [64]. A review of seven international studies confirmed the growing role of regression, time series and ML methods in strengthening supply chain resilience by consistently improving forecasting accuracy, reducing waste and optimizing resource allocation [65]. Emerging tools such as the American Red Cross's ‘Clara’ chatbot illustrate how conversational AI can further improve donor communication and streamline appointment scheduling [66].

Together, these cases demonstrate a continuum of success—from basic digital interventions to integrated AI systems—showing that digital solutions, regardless of complexity, can strengthen donor engagement and maintain blood supply. They also provide a roadmap for blood banks: start with low‐cost, high‐impact tools such as SMS alerts or chatbots; advance to predictive algorithms for donor management, inventory optimization and transfusion safety; and ultimately adopt integrated, data‐driven platforms that leverage electronic health records and supply chain analytics. This step‐by‐step, capacity‐based approach ensures measurable benefits while laying the foundation for more advanced systems.

## CONCLUSION

The integration of AI into health care, and more specifically into blood donor management systems, holds significant potential to transform and enhance operational efficiency, donor engagement and safety. Techniques such as RPA for the automation of repetitive tasks, big data analysis for the prediction of blood demand and donor behaviour and AI‐driven chatbots can optimize donor communication and retention and ensure a safe and sustainable blood supply. Emerging applications, such as rare donor identification and management, further highlight the value of AI in addressing critical gaps within transfusion medicine. However, with these advancements come challenges related to data privacy, algorithmic transparency and the ethical considerations of AI deployment. Ensuring the responsible use of AI while balancing innovation with regulatory compliance and sustainability—together with careful attention to practical implementation—will be crucial in maximizing the benefits of AI for both blood donation systems and the broader healthcare industry. Further research and investment in AI technologies are needed to continue refining these systems and ensure that they are implemented effectively and ethically.

## CONFLICT OF INTEREST STATEMENT

The author declares no conflicts of interest.

## Data Availability

Data sharing not applicable to this article as no datasets were generated or analysed during the current study.
